# Allelic imbalance studies of chromosome 9 suggest major differences in chromosomal instability among nonmelanoma skin carcinomas

**DOI:** 10.1590/S1516-31802004000100005

**Published:** 2004-01-08

**Authors:** Gomes Gabriela Pereira, Moraes Aparecida Machado, Stoff Hamilton Ometto, Laura Sterian Ward

**Keywords:** Allelic imbalance, Basal cell neoplasms, Skin cancer, Loss of heterozygosity, Desequilíbrio alélico, Basocelular, Câncer da pele, Perda de heterozigosidade, Genética

## Abstract

**CONTEXT:**

Loss of heterozygosity in the 9p21-p22 region, has been frequently described in a wide range of human malignancies, including familial melanomas. Also, losses and gains in other regions of chromosome 9 have frequently been observed and may indicate additional mechanisms for basal cell tumorigenesis.

**OBJECTIVE:**

To investigate allelic imbalance in the 9p21-p22 region, among basal cell carcinomas.

**TYPE OF STUDY:**

Microsatellite analysis.

**SETTING:**

Two dermatology services of public universities in São Paulo and the Laboratory of Cancer Molecular Genetics of Universidade Estadual de Campinas (Unicamp).

**PARTICIPANTS:**

13 patients with benign skin lesions consecutively referred to the outpatient dermatology clinics of Unicamp and Universidade Estadual de São Paulo (Unesp) and 58 with malignant skin tumours.

**MEAN MEASUREMENTS:**

We examined 13 benign cases including four of solar keratosis, three keratoachanthomas, three melanocytic nevi, two of Bowen's disease and one of neurofibroma, and 58 malignant skin tumors: 14 of squamous cell, 40 basal cell carcinomas and four melanomas. Participating patients had the main tumor and a normal portion of non-adjacent skin surgically removed. DNA was extracted from the tumor and matching normal tissue. We used four sets of primers to amplify polymorphic microsatellite repeats on chromosome 9, two of them targeting the 9p21-p22 region.

**RESULTS:**

We identified eight cases (20%) of allelic imbalance among basal cell carcinomas, two cases of loss of heterozygosity and six cases of microsatellite instability in the 9p21-p22 region. Additional markers were also involved in three of these tumors. No events were detected among the benign or the other malignant cases.

**CONCLUSION:**

This phenotype dependency suggests that there is a major distinction between the two most important forms of nonmelanoma skin cancers in their tendency to present microsatellite instability in chromosome 9. Since the *CDKN2a/ p16INK4a, p19ARF* and *p15INK4b* tumor suppressor genes do not appear to be responsible for the observed abnormalities, other genes at 9p21-p22 may be involved in the pathogenesis and progression pathway of basal cell carcinomas.

## RESUMO

**CONTEXTO:**

Vários estudos de perda de heterozigozidade na região 9p21-p22, que abriga os genes supressores tumorais CDKN2a/ p16INK4a, p19ARF e p15INK4b, têm sido realizados em uma ampla série de tumores humanos, incluindo os melanomas familiares. Perdas e ganhos em outras regiões do cromossomo 9 também têm sido observados com freqüência e podem indicar mecanismos adicionais no processo de tumorigênese dos carcinomas basocelulares da pele.

**OBJETIVO:**

Investigar o equilíbrio alélico existente na região 9p21-p22 em carcinomas basocelulares.

**TIPO DE ESTUDO:**

Análise molecular de marcadores de microssatélites em tumores e controles.

**LOCAL:**

Dois serviços de dermatologia de atendimento terciário em universidades públicas de São Paulo e o Laboratório de Genética Molecular do Câncer da Universidade Estadual de Campinas (Unicamp), Brasil.

**PARTICIPANTES:**

Examinamos 13 casos benignos, incluindo 4 queratoses solares, 3

queratoacantomas, 3 nevos melanocíticos, 2 doenças de Bowen e 1 neurofibroma cutâneo, além de 58 tumores malignos da pele: 14 de células escamosas, 40 carcinomas basocelulares e 4 melanomas; em pacientes consecutivamente encaminhados à clínica de Dermatologia da Unicamp e que concordaram em participar do estudo.

**VARIÁVEIS ESTUDADAS:**

O tumor principal e uma porção normal de pele não-adjacente foram removidos cirurgicamente de pacientes que consecutivamente procuraram os ambulatórios de dermatologia da Universidade Estadual de Campinas (Unicamp) e da Universidade Estadual de São Paulo (Unesp), São Paulo, por causa de lesões cutâneas. Extraímos DNA tanto de tecido tumoral como do correspondente tecido normal de cada paciente. Para amplificar regiões de repetição polimórfica de microssatélites do cromossomo 9, foram utilizados quatro pares de primers, sendo dois deles destinados à região 9p21-p22.

**RESULTADOS:**

Identificamos oito casos (20%) de desequilíbrio alélico entre os carcinomas basocelulares, sendo dois casos de perda de heterozigozidade e seis casos de instabilidade de microssatélite na região 9p21-p22. Outros marcadores também mostravam anormalidades em três destes tumores, enquanto nenhuma alteração foi detectada entre os casos benignos e nos outros tumores malignos.

**CONCLUSÃO:**

Esta dependência fenotípica sugere que existem diferenças importantes no comportamento das formas mais comuns de tumores cutâneos não-melanocíticos em relação à sua tendência para instabilidade de microssatélite no cromossomo 9. Considerando-se que os genes CDKN2a/p16INK4a, p19ARF e p15INK4b não parecem responsáveis pelas anormalidades observadas, outros genes em 9p21-p22 podem estar envolvidos na etiopatogenia e na progressão dos carcinomas basocelulares.

## INTRODUCTION

Nonmelanoma skin cancer is the most common malignancy in human beings, and its incidence continues to increase.^1,2^ Skin cancers, like other human tumors, arise from a single transformed cell that presumably gained growth advantage through damage to the genes that control cell proliferation.^3^ The neoplastic clone, in which proliferative controls are disrupted, may also have a higher propensity to develop further genetic abnormalities.^4^ As the genome becomes unstable, the cell is more prone to develop large-scale chromosome aberrations, amplifications, deletions and translocations. The consequent accumulation of genetic abnormalities is associated with increasing genetic heterogeneity in the tumor clone.^5-7^

Genome instability has been characterized in the majority of human tumors as chromosomal instability (aneuploidy, deletions or amplifications).^7^ Tumor groups exhibit mutations on a smaller scale, such as nucleotide instability,^8^ minisatellite instability^9^ or microsatellite instability.^10^ Microsatellite instability is manifested by small deletions or expansion in the copy number of tandem repeat sequences in tumor DNA, as compared with matching normal DNA.^10^ First discovered in hereditary nonpolyposis colorectal cancer syndrome, which is one of the most common syndromes associated with cancer predisposition in man, microsatellite instability has been implicated in about 15% of sporadic colorectal cancers, as well as in cancers at several other sites such as the bladder and stomach.^10-12^

Numerous observations have shown that lterations in microsatellite DNA, although random, tend to be associated with the mutation of genes that control cell growth and apoptosis, and such modifications at microsatellite loci seem to play an important role in the development of human cancers.^12^

Because karyotype abnormalities have frequently been reported in melanocytic lesions and a high frequency of heterozygosity loss has been demonstrated in chromosome 9, it has been proposed that this chromosome contains one or more tumor suppressor genes that may also be involved in squamous cell and basal cell carcinomas.^13-17^ p16INK4a is a tumor suppressor protein encoded by the CDKN2A gene in the 9p21 region, a gene that can generate different transcripts. It functions as an inhibitor of the D-type cyclin-dependent kinases 4 and 6 (CDK4 and CDK6) that are involved in cell cycle regulation. Inactivation of p16 is a frequent event in epidermoid squamous cell cancers in general and has been related to familial melanoma, but its role in basal cell skin carcinomas is still unclear.

The objective of this study was to investigate the role of allelic imbalance in human chromosome 9p21-p22, in basal cell carcinomas, in comparison with a series of sporadic benign and malignant skin lesions.

## METHODS

The study was approved by the Ethics Committee of the University Hospital of the Faculdade de Medicina da Universidade Estadual de Campinas (Unicamp), and informed written consent was obtained from a total of 71 patients. Thirteen presented benign lesions and 58 had malignant skin tumors. The benign cases included four solar keratosis, three keratoachanthomas, three melanocytic nevi, two cases of Bowen's disease and one neurofibroma. The malignant skin tumors included 14 squamous cell (8 males and 6 females, aged 52 ± 12 years old), 40 basal cell carcinomas (20 males and 20 females, aged 54 ± 16 years old) and four melanomas (2 males and 2 females, aged 51 ± 14 years old). All patients were carefully examined and previous medical conditions were particularly considered, especially organ transplantation, immunosuppressive therapy, other malignancies and HIV infection.

DNA from a central part of the tumor and DNA from normal adjacent skin tissue samples were isolated immediately after surgical resection, according to standard methods, using proteinase K digestion and phenol-chloroform extraction. The DNA concentration was determined by means of 260/280 spectrophotometry. Two sets of primers obtained from Research Genetics (Huntsville, Alabama) were used to amplify the D9S200 and D9S1748 loci at 9p12- p21. Another pair of primers targeted the 9q31-34 region (D9S120) and, finally, one pair of primers targeted the 9p22-23 region (D9S156). Primer sequences were extracted from the Genome Database via the internet (http://www.gdb.org). The polymerase chain reaction (PCR) was performed using 20-µl volumes of a mixture containing 30-50 ng of DNA, 50 nm of each primer, 10 mM tris-HCl (pH 8.0), 50 mM MgCl_2_, 100 uM of each dinucleotide triphosphate and 0.5 U Taq of DNA polymerase. The MgCl_2_ concentrations ranged from 1 to 1.75 mM and the annealing temperatures varied from 56 to 62° C. Amplifications were carried out for 35 cycles of 94° C for 45 seconds, 56-62° C for 45 seconds and 72° C for 1 min, with an initial denaturation step of 94° C for 2 min and a final extension step of 72° C for 7 min using a Perkin-Elmer 9600 GeneAMp PCR system. The PCR products were mixed with loading buffer (95% formamide, 20 mM EDTA, 0.05% bromophenol blue, 0.05% xylene cyanol) and 6 µl of this mixture was combined with 6 µl of heat-denatured formamide. This was run overnight at 65 V, on 8% polyacrylamide, 7-M urea gel stained with silver nitrate. Informative patients were identified and the allelic loss was scored using the GeneScan software (Kodak).

## RESULTS

All 71 cases were informative for at least one of the primers used to examine the 9p21-p22 chromosome region. Cases were scored as showing allelic imbalance if the ratio between the two alleles in the tumor was 50% less than what was detected for the normal sample. A significant reduction of more than 65% of the signal intensity of one of the two tumor alleles was recorded as loss of heterozygosity (although this does not formally exclude allelic imbalance secondary to amplification of one allele). Microsatellite instability was defined by the presence of bands that were absent from the normal sample. Homozygous cases were considered not informative for loss of heterozygosity.

**Figure 1:**
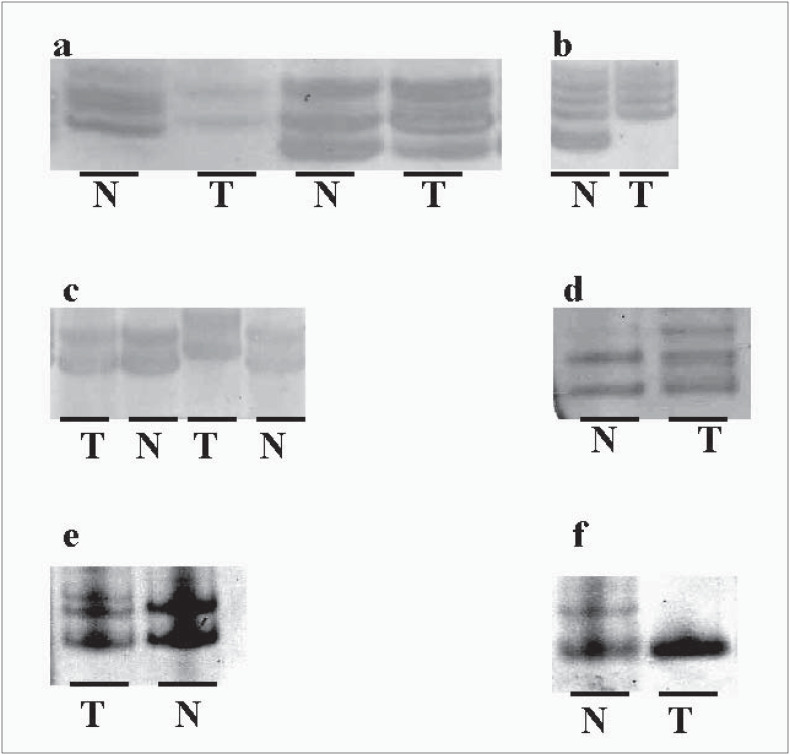
Examples of allelic imbalance. Panel a shows allelic imbalance of the tumor (allelic ratio 75% less than the corresponding normal alleles) in the first pair of samples and a loss of heterozigosity in the third pair of basal cell carcinoma samples tested with the D9S1748 marker. Panel b demonstrates that the latter tumor exhibits an extra band on 9q31-34, using the D9S120 marker. Panels c and d show basal cell carcinoma samples presenting microsatellite instability and loss of heterozigosity at 9q12-21, using the D9S200 and D9S1748 markers, respectively.

Two basal cell carcinomas presented heterozygosity loss (Figures 1a and 1d) and six basal cell carcinomas showed an extra band with one of the microsatellite markers for the 9p21 region (Figure 1c). Three of these six basal cell carcinoma patients also showed allelic imbalance in the 9p23-p22 region and two of them additionally presented an extra band at the 9q31-q34 locus, thus indicating replication errors (Figure 1b ). All of these eight patients were white and presented multiple skin lesions, particularly at sun-exposed sites. The tumors examined were excised from the head/neck region of five patients and from the chest and back, respectively, of three other patients. We were not able to identify any clinical pattern that could differentiate these cases from the remaining basal cell patients.

## DISCUSSION

Tumors arise from a series of selection pressures that favor growth and survival.3 A large proportion of skin cancers, the most common malignancies in the Western world, are of nonmelanoma type. Basal cell carcinomas account for more than 75% of cases and squamous cell carcinomas for a further 20%.^1,2,18-20^ Epidemiological studies suggest that sunlight (particularly ultraviolet-B radiation, UVB) is a strong risk factor for the appearance of basal cell carcinoma, although other factors are also involved.^19,20^ Squamous cell carcinomas of the skin also are associated with exposure to UV light and both basal cell and squamous cell carcinomas are derived from the same cell type, the keratinocyte cell. However, in contrast with squamous cell carcinoma, basal cell carcinomas are slow-growing, locally invasive tumors that rarely produce metastasis.^19,20^

Microsatellite instability has been identified in a wide variety of human tumors, both familial and sporadic. It is associated with early or late stages of tumor progression, and a higher frequency of microsatellite instability has been noted in certain tumor subtypes.^10,12^ Using 71 paired samples of normal and tumor DNA, amplified by PCR for the analysis of polymorphic dinucleotide repeats on chromosome 9, we obtained a 20% rate of allelic imbalance in basal cell carcinomas. In contrast, there were no such events in lesions due to squamous cell carcinoma or malignant melanomas, or in benign lesions. In agreement with our data, a recent study on basal cell car- cinomas using comparative genomic hybridi- zation has demonstrated recurrent chromo- somal gains at 9p in 20% of the cases, which was coincidentally the same rate of allelic im- balance we obtained.^21^

We found evidence of frequent chromosomal instability in the 9p21-p22 region that harbors the CDKN2a/p16INK4a, p19ARF and p15INK4b tumor suppressor genes. Loss of p16 function is associated with increased susceptibility to a variety of malignancies, and germline mutations of p16INK4a have been found in up to 20-30% of melanoma-prone families.^22-24^ The INK4a-ARF locus also plays an important role in sporadic nonmelanoma skin cancers. p16INK4a UV-induced muta- tions (CC:GG > TT:AA tandem transition or C:G > T:A transition at dipyrimidine sites) have been described in up to 12% of sporadic skin carcinomas and seem to occur independ- ently from *p53* mutations.^24^

Some early data reported low frequencies of 9p loss and p16 mutations in basal cell carcinomas, in contrast to findings from squamous cell carcinomas.^14,25^ More recently, Saridaki et al., although reporting a lack of mutations of p16INK4a/p19ARF among Greek patients with basal cell carcinoma of the skin, found similarly high frequencies of heterozygosity loss at the 9p21-p22 region in basal cell and squamous cell carcinomas.^26,27^ These data contrast with ours, and could reflect a particular genetic pattern related to our population.

With regard to squamous cell carcinomas, Kushida et al., studying Japanese patients, found no microsatellite instability in 37 actinic keratosis and 14 sporadic squamous cell cases studied using six microsatellite markers. Loss of heterozygosity was found in 7 actinic keratosis cases, but in only one case of squamous cell carcinoma, thus leading these authors to conclude that actinic keratosis was not likely to proceed to squamous cell carcinomas in such patients.^28^ On the other hand, there has been recent evidence that the loss of p16(INK4a) is implicated in the progression of actinic keratosis lesions to squamous cell carcinomas, at least among Caucasians.^29^ Microsatellite instability has been reported in keratoacanthoma patients, but at a low frequency that suggests that these genetic events are uncommon in sporadic lesions.^30,31^

Three of our basal cell carcinoma cases presented instability in two or more markers of chromosome 9, thus suggesting the existence of a subgroup of patients that may be more prone to presenting allelic imbalance or replication errors. Unfortunately, we were unable to identify any particular pattern that could differentiate such patients from the remaining basal cell cases.

## CONCLUSIONS

The fact that only basal cell carcinomas presented allelic imbalance suggests that there is a major distinction between the two most important forms of nonmelanoma skin cancers in their tendencies to present microsatellite instability in chromosome 9. This is likely to result from a fundamental difference in the mechanisms controlling chromosomal stability in these two forms of cancer and demonstrates that basal cell carcinomas may be more genetically unstable than previously thought.

Since the CDKN2a/p16INK4a, p19ARF and p15INK4b tumor suppressor genes do not appear to be responsible for the abnormalities observed,^26^ other genes at 9p21-p22 may be involved in the pathogenesis and progression pathway of basal cell carcinomas.
